# Identification of common carp (*Cyprinus carpio*) microRNAs and microRNA-related SNPs

**DOI:** 10.1186/1471-2164-13-413

**Published:** 2012-08-21

**Authors:** Ya-Ping Zhu, Wei Xue, Jin-Tu Wang, Yu-Mei Wan, Shao-Lin Wang, Peng Xu, Yan Zhang, Jiong-Tang Li, Xiao-Wen Sun

**Affiliations:** 1 The Centre for Applied Aquatic Genomics, Chinese Academy of Fishery Sciences, Beijing, 100141, China; 2 College of Fisheries and Life Science, Shanghai Ocean University, Shanghai, 201306, China; 3 Department of Psychiatry and Neurobiology Science, University of Virginia, Charlottesville, VA, 22911, USA

**Keywords:** miRNAs, Targets, SNPs, miRNA biogenesis, Common carp

## Abstract

**Background:**

MicroRNAs (miRNAs) exist pervasively across viruses, plants and animals and play important roles in the post-transcriptional regulation of genes. In the common carp, miRNA targets have not been investigated. In model species, single-nucleotide polymorphisms (SNPs) have been reported to impair or enhance miRNA regulation as well as to alter miRNA biogenesis. SNPs are often associated with diseases or traits. To date, no studies into the effects of SNPs on miRNA biogenesis and regulation in the common carp have been reported.

**Results:**

Using homology-based prediction combined with small RNA sequencing, we have identified 113 common carp mature miRNAs, including 92 conserved miRNAs and 21 common carp specific miRNAs. The conserved miRNAs had significantly higher expression levels than the specific miRNAs. The miRNAs were clustered into three phylogenetic groups. Totally 394 potential miRNA binding sites in 206 target mRNAs were predicted for 83 miRNAs. We identified 13 SNPs in the miRNA precursors. Among them, nine SNPs had the potential to either increase or decrease the energy of the predicted secondary structures of the precursors. Further, two SNPs in the 3’ untranslated regions of target genes were predicted to either disturb or create miRNA-target interactions.

**Conclusions:**

The common carp miRNAs and their target genes reported here will help further our understanding of the role of miRNAs in gene regulation. The analysis of the miRNA-related SNPs and their effects provided insights into the effects of SNPs on miRNA biogenesis and function. The resource data generated in this study will help advance the study of miRNA function and phenotype-associated miRNA identification.

## Background

MicroRNAs (miRNAs) are endogenous small non-coding RNA molecules that are an average of 22 bp long [1]. They exist widely in metazoa, viridiplantae and viruses [2-7] and play essential roles in gene expression regulation by binding to their target genes, leading to translational repression or transcript degradation [8,9]. The role of miRNAs in the regulation of genes leads to their involvement in diverse biological processes that include animal organ development and growth [10], cell differentiation and proliferation [11], innate and adaptive immunity [12], and signal transduction [13]. Many studies have identified miRNAs in fish species [14-18]. After miRNAs have been identified in a species, one of the main aims is to identify the miRNA target genes. Computational predictions have been widely applied to miRNA target identification [19]. Common carp is one of the main commercial fishes captured and cultured worldwide. Its agricultural production accounts for nearly 30% of fresh water fish cultured in China. However, miRNAs targets have not been investigated in common carp.

MiRNAs bind to their target genes based on sequence complementarity. Mutations in miRNAs or in their target sites have been found to either create or disturb miRNA-target interactions. Many studies have reported the effect of SNPs in the 3’untranslated regions (3’UTRs) of the target genes. For example, in Texel sheep, a SNP in the 3’UTR of *GDF8* created a binding site targeted by two miRNAs, miR-1 and miR-206, resulting in *GDF8* inhibition and increased muscular hypertrophy [20]. In contrast, a SNP in the 3’UTR of the *SPL14* gene in rice perturbed osa-miR156-associated translational regulation which led to an improved rice plant with reduced tiller number, better gain yield and enhanced lodging resistance [21]. In addition, SNPs in miRNAs have been shown to affect miRNA regulation resulting in phenotypic changes [20,22,23]. For instance, in Chinese Holstein cattle, a SNP in bta-miR-484 disrupted miRNA binding which relieved the transcriptional repression and increased the expression of the target gene, the heat-shock transcription factor 1 [24]. Moreover, SNPs in miRNAs can also affect miRNA biogenesis. Two mutations in the seed region of hsa-miR-96 impaired the biogenesis of the miRNA and result in a significant reduction of mRNA targeting [25]. Because of the recently recognized significance of SNPs in miRNA biogenesis and regulation, many reports have concentrated on collecting miRNA-related SNPs and investigating their influence on miRNA function [20,26-28]. However, all these studies have focused on model species mainly because a substantial amount of information on miRNAs and SNPs is available for these species. For common carp, no reports of miRNA-related SNPs and their effects have been published so far.

In this study, we used a combinational strategy to identify miRNAs in common carp and characterized them based on their conservation and expression profiles. Next, we used target prediction software to predict the miRNA targets. After identifying the potential targets we scanned SNPs in the miRNAs and studied their effects on miRNA biogenesis and target alteration. Finally, we identified SNPs in targets’ 3’UTRs and predicted the influence of the identified SNPs on miRNA regulation of the target genes.

## Results

### Identification of common carp miRNAs with computational prediction and small RNA-sequencing

The previously published high-quality (Q20) BAC end sequences (BES) [29] that were assembled into 38,883 genomic contigs, together with 49,669 common carp transcriptome contigs [30], were used as reference sequences for miRNA identification.

To identify common carp miRNAs, we firstly performed a homology-based prediction. The prediction pipeline is shown schematically in Additional file 1: Figure S1. We downloaded 16,822 animal miRNAs from miRBase 17.0 [31]. The analysis of the hairpin structures of animal miRNA precursors using UNAfold [32] showed that over 96.53% of miRNA precursors satisfied the criteria for hairpin structures previously described by Fu et al. [17] [see Additional file 2: Figure S2. We identified 9,656 non-redundant miRNAs after removing identical miRNAs. After aligning the unique miRNAs to the reference sequences and carefully evaluating the hairpin structures, 81 conserved miRNAs were identified from common carp.

**Figure 1:**
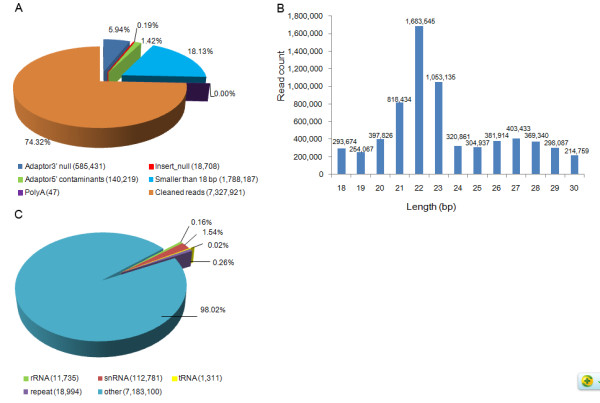
**Analysis of sequencing reads of common carp. A**: Summary of sequencing reads. **B**: The length distribution of cleaned sequencing reads. **C**: The cleaned reads were blasted against the Rfam, common carp ribosomal RNAs collected from GenBank, and common carp repeats to annotate rRNA, tRNA, snRNA, and repeats.

Next, a small RNA (sRNA) library was constructed from multiple tissues of 17 adult common carp and sequenced following the illumina protocol [28]. A total of 11,665,437 raw reads were generated and 7,327,921 cleaned reads (62.82%) were obtained and used in the analysis (Figure 1A). The length of the cleaned reads peaked at 22 bp (Figure 1B). Using BLASTN searches, a total of 125,827 cleaned reads (1.72% out of clean reads) were identified as fragments of other sRNAs (rRNA, tRNA and snRNA) and another 18,994 reads (0.26% out of clean reads) aligned to common carp repeats (Figure 1C). These were removed from the dataset and the remaining 7,183,100 reads (98% out of clean reads) were used for miRNA identification with MIREAP. A total of 68 sRNAs were identified as reliable miRNAs. Among them, 36 miRNAs were homologous to known animal miRNAs and the precursors of an additional 11 miRNAs could be aligned to the NCBI non-redundant nucleotide database using BLASTN with an e-value of 1e-10. The remaining 21 miRNA had no hits, indicating that they might be common carp specific.

Finally, the results from the two methods were integrated into a non-redundant dataset that included 92 conserved miRNAs and 21 specific miRNAs. The length distribution of the miRNAs was between 20 and 26 bp. Detailed information about the predicted miRNAs, including the prediction method, conservation, reference sequences, location in the reference sequences, precursor sequences, hairpin structures, minimum folding free energies, mature sequences and A + U content is available in Additional file 3: Table S1, Additional file 4: Table S2, Additional file 5: Table S3, and Additional file 6: Table S4.

To validate the reliability of the predicted miRNAs, their expression in the RNAs from the pooled-tissues was examined by PCR. Ten miRNAs, including conserved and specific miRNAs [see Additional files 7: Table S5], were selected randomly from the dataset. The PCR results showed that all the selected miRNAs could be amplified [see Additional file 8: Figure S3], indicating that these miRNAs were correctly identified and truly expressed.

### Characterization of common carp miRNAs

Many miRNAs are often located close to each other, forming gene clusters that have a common transcription promoter [33]. We discovered five miRNA clusters (Table 1) among the common carp miRNAs. Four clusters consisted of conserved miRNAs while one cluster had one conserved miRNA and one specific miRNA. Interestingly, three of the clusters contained miRNAs from the mir-430 family. In the zebrafish genome (Zv_9), these three clusters are located close together, suggesting that they might also be part of one bigger miRNA cluster in the common carp genome. We looked for members of the mir-430 family in other animals and found that this family existed only in zebrafish, medaka and sea lamprey. This result indicates that the mir-430 family might be fish specific. Further experiments are warranted to study the function of this family of miRNAs.

**Table 1:** Common carp miRNA clusters

**Cluster**	**Members**	**Gene family**	**Locations**	**Strand**	**Cluster length(bp)**	**Zebrafish genomic region**
1	miR-212,	mir-132	CYC023A01I23/2:200-591*	-	392	chr15: 25232199- 25232621
	miR-132					
2	miR-430b-2	mir-430	CYC084B02N10/1:362-723	-	362	chr4: 28007374- 28007937
	miR-430c-2					
3	miR-430b-1	mir-430	utg7180000000224:174-688**	+	515	chr4: 28012479- 28013329
	miR-430a,					
	miR-430c-1					
4	miR-430a,	mir-430	utg7180000000234:306-1206	+	901	chr4: 28001389-28010326
	miR-430c-1					
	miR-430b-1					
5	miR-7,		utg7180000001602:502-775	-	274	chr5:56984908- 56985224*
	s0021					

*:“CYC” presents unassembled BESs.

**: “utg” presents assembled BESs.

**Figure 2:**
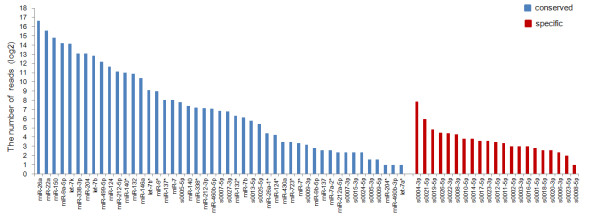
**Expression levels of common carp miRNAs.** The expression level of one miRNA is calculated as the number of sequencing reads aligned to it.

The sRNA sequencing allowed us to identify miRNAs and also to determine the expression levels of the miRNAs [15,28]. The number of reads that could be aligned to each of the miRNAs was assumed to represent the expression level of the miRNA. We found a lot of divergence in the abundance of the different miRNAs (Figure 2). In general, the conserved miRNAs had higher expression than the specific miRNAs (Mann Whitney test, P value = 7.91e-3). This result is consistent with previous observations that non-conserved miRNAs are often expressed at lower levels than miRNAs with tissue-specific or developmental-specific expression patterns [34-36]. The seven most abundant miRNAs, each with over 8,000 reads, were conserved miRNAs. These results agree with the conclusion that evolutionarily conserved miRNAs are often the most abundant [37]. In contrast, miR-204*, miR-406b* and let-7a* had extremely low frequency in our library (frequency = 2), consistent with the observation that most miRNA*s showed weak expression and that their expression levels were much lower than their corresponding miRNAs [28]. This is because miRNA*s are rapidly degraded during the biogenesis of mature miRNAs [28].

**Figure 3:**
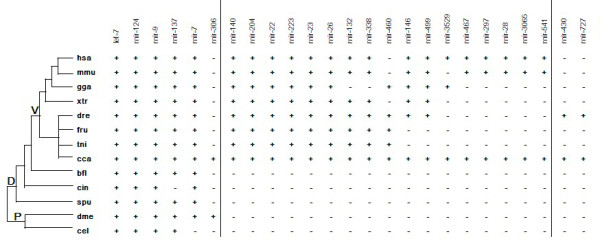
**Conserved miRNAs in common carp.** The presence of miRNA is indicated by plus (+); the absence of miRNA is indicated by minus (−). Abbreviations: hsa—*Homo sapiens*; mmu—*Mus musculus*; gga—*Gallus gallus*; xtr—*Xenopus tropicalis*; dre—*Danio rerio*; fru—*Takifugu rubripes*; tni—*Tetraodon nigroviridis*; cca—*Cyprinus carpio*; bfl—*Branchiostoma florida*; cin—*Ciona intestinalis*; spu—*Strongylocentrotus purpuratus*; dme—*Drosophila melanogaster*; cel—*Caenorhabdits elegans*; P—Protostomia; D—Deutostomia; V—Vertebrata.

The conservation of miRNAs has been used to study miRNA phylogenetic evolution [33]. Twenty-one conserved miRNA families were clustered into three groups based on their phylogenetic distributions (Figure 3). Six miRNA families (mir-124, mir-9, mir-137, mir-7, mir-306 and let-7) were shared by both protostomes and deuterostomes; 17 miRNAs families were present only in vertebrates; and the remaining two miRNA families (mir-430 and mir-727) were identified only in fish and were possibly fish-specific miRNAs. Six miRNA families (mir-3529, mir-467, mir-297, mir-28, mir-3065 and mir-541) were first detected in fish, but have been identified in other species.

### Expression profile of miRNAs among the developmental stages

**Figure 4:**
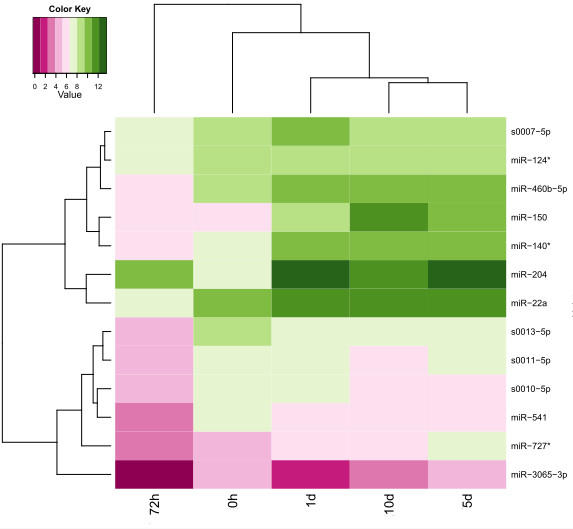
**The heatmap of identified miRNAs during common carp developmental stage.** There are 14 rows and 5 columns corresponding to each miRNA and developmental stage, respectively. The heatmap was drawn on log 2 normalized expression of each miRNA in relation to the expression of U6. 0 h, 72 hpf, 1 dph, 5 dph and 10 dph stand for fertilized oocytes, 72 hours post-fertilization embryos, 1 day post-hatching larva, 5 day post-hatching larva and 10 day post-hatching larva.

The expression of homologous miRNAs in other species may help us infer the expression of the common carp miRNAs. To study the expression pattern of miRNAs during the embryo development, we selected eight miRNAs of which homologous miRNAs were related to embryo development [38-42]. Another four common carp specific miRNAs were also randomly selected. We found that the expression patterns of some of the miRNA families were associated with certain development stages. Hierarchical clustering of the RT-qPCR products showed three major expression patterns: a) fertilized oocytes expression (0 h) which might be related to maternal miRNAs; b) embryonic expression (72 hpf) during hatching; and c) larvae expression (1 dph, 5 dph and 10 dph) when the larvae undertake exogenous feeding (Figure 4).

The expression levels of all the miRNAs varied among the developmental stages. The expression levels decreased from the oocytes stage to embryonic stage and then increased again in the larvae stage. At the embryonic stage, the expression of the majority of miRNAs was lower than at any of the other stages. The most dominant miRNAs at the embryonic stage were from the miRNA-204 family. The miR-204 family has been reported to be associated with mouse eye development [43], indicating that this miRNA family may play roles in organogenesis in the common carp embryo.

Hierarchical clustering also indicated that the miRNAs selected for RT-qPCR were classified into two groups based on their expression patterns. In general, the expression of miRNAs in the first group (s0007-5p, miR-124-5p, miR-460b-5p, miR-150, miR-140-3p, miR-204, and miR-22a) was higher than the expression of the miRNAs in the second group (s0013-5p, s0011-5p, s0010-5p, miR-541, miR-727-5p, and miR-3065-3p). The similar expression patterns of the miRNAs in each of the groups may imply that they share similar functions, which would help us understand the function of the specific miRNAs based on the known function of other miRNAs in the same group.

### Prediction of miRNA targets

To predict miRNA target genes in common carp, we downloaded 506 mRNAs containing 3’UTR information from GenBank [44] and used a combination of TargetScan [45] and PITA [46] to predict miRNA targets. We identified 394 miRNA-binding sites in 206 mRNAs targeted by 83 miRNAs; an average of 2.5 mRNAs per miRNA. The predicted target genes were found to be involved in a broad range of biological functions; for instance, transferase activity, hydrolase activity, nucleotide binding, protein binding and signal transducer activity [see Additional file 9: Table S6. Strikingly, *IGF-IRb* [GenBank: AY144592] had the most miRNA binding sites; 13 sites targeted by 12 miRNAs.

Furthermore, we found 18 miRNAs that were generated from the antisense strands of 19 mRNAs. The miRNAs were perfectly complementary to the sense mRNAs [see Additional file 10: Table S7. MiRNAs in such arrangements might function to depress sense mRNA expression or to disrupt mRNA splicing [47].

**Figure 5:**
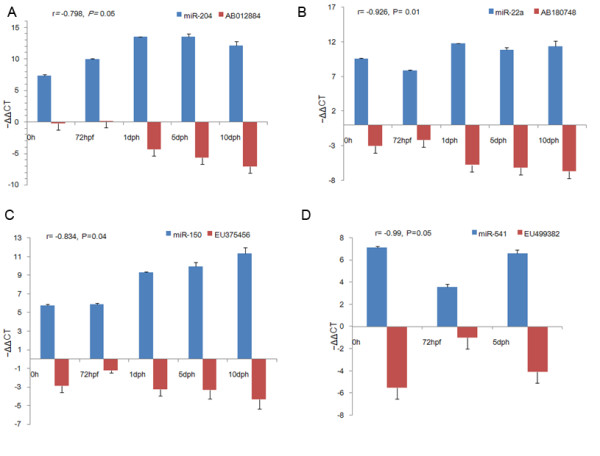
**The expression of four miRNA-target pairs during five developmental stages in common carp.** Expression levels of miRNAs and their predicted targets were detected by RT-qPCR at five developmental stages (X axis). Y axis shows the expression levels of miRNA and its target. The expression of each miRNA was normalized to U6 and then transformed to a log 2 scale. The expression of each target gene was relative to beta-actin and also transformed to a log 2 scale. The value of r is the correlation coefficient. All four miRNA-target pairs showed significantly reciprocal expression patterns (p ≤ 0.05). Developmental stages are specified in Figure 4 legend.

To investigate the regulation of predicted targets by miRNAs, the expression of four miRNA-target pairs was examined by reverse transcribed-quantitative real-time PCR (RT-qPCR) analyses during five developmental stages. All four pairs showed significantly reciprocal expression patterns (Figure 5 A-D), consistent with the observation that miRNAs in mammalian predominantly function to decrease target gene levels [48].

### Influence of SNPs in miRNAs on the energy of the miRNA secondary structure

SNPs in precursors have been reported to enhance or interrupt miRNA biogenesis [49,50]. We identified 13 SNPs in seven precursors by mapping the cleaned sRNA sequencing reads to the precursors. These SNPs were classified into four types: 1) two SNPs in the mature miRNA; 2) six SNPs in the stem regions; 3) three SNPs in the loops; and 4) two SNPs in the anti-stem regions (Table 2).

**Table 2:** The effect of SNPs on miRNA precursors energy change

**Precursor**	**SNP position in precursor**	**SNP**	**SNP location**	**△△G (kcal/mol)**
s0008	19	A → C	mature	−0.3
s0027-1	53	U → G	mature	−1.4
s0008	39	C → G	Stem	2.2
s0009	41	U → C	Stem	−0.5
s0015	48	C → A	Stem	0.3
s0015	50	U → C	Stem	−1.3
s0027-1	52	G → U	Stem	0.6
s0007	43	G → A	Stem	−0.1
mir-140	43	G → A	Loop	0
s0007	39	A → C	Loop	0
s0009	45	U → G	Loop	0
s0013	40	C → U	anti-stem	2.7
s0013	41	A → G	anti-stem	1

MFE: minimum free energy.

ΔΔG: the MFE difference value between SNP-type precursors and wild-type precursors. The minus value indicated that the SNP-type precursors had lower structure energy than the wild precursors. Otherwise, the former had higher structure energy than the latter.

In addition, we PCR-amplified and resequenced five miRNA precursors surrounding nine putative SNPs by conventional Sanger sequencing. All the regions were successfully covered. When the Sanger reads were compared with the reference precursors, we found that seven SNPs were successfully detected with Sanger sequencing [see Additional file 11: Figure S4].

Next, we investigated the effect of the SNPs on the energy change (ΔΔG) of the secondary structures of the precursors. We found that SNPs in the loop regions did not change the energy of the structure while the other 10 SNPs (types 1, 2 and 4) did change the energy of the predicted secondary structures. For nine of the SNPs, the absolute energy change values were ≥ 0.3 kcal/mol, the minimum energy change reported to be required to change the production of mature miRNAs [49]. Gong et al. summarized the rule that if a SNP decreases the hairpin structure energy, the production of the mature miRNA will be reduced; and if the SNP increases the energy, the production of the mature miRNA will increase [51]. The SNPs reported here might therefore enhance or reduce the production of the mature miRNAs.

### MiRNA-mRNA interaction alteration by SNPs in targets

SNPs in the miRNA binding site can alter the miRNA-mRNA interaction [23]. To identify SNPs in common carp mRNA 3’UTRs, we aligned the 298,817 trimmed 454 transcriptome reads to 506 mRNAs containing 3’UTR annotation and identified 464 SNPs in 95 mRNAs. A total of 57 SNPs were located in the 3’UTRs of 33 mRNAs. These mRNAs were called SNP-type mRNAs. In addition, we PCR-amplified and resequenced ten randomly selected SNPs in nine mRNA 3’UTRs. A comparison between the Sanger sequences and the reference 3’UTRs showed that nine SNPs were successfully detected by conventional sequencing [see Additional file 12: Figure S5.

Fifteen SNP-type mRNAs were predicted to have 35 sites targeted by 27 miRNAs. Most of these binding sites existed in both the wild type mRNAs and SNP-type mRNAs. However, one SNP in the 3’UTR of *C1rs-A* [GenBank: AB042609] was predicted to result in target gain and one SNP in the 3’UTR of *cyp c1.02* [GenBank: AJ292212] was predicted to lead to target loss (Table 3). These two SNPs were also confirmed by Sanger sequencing.

**Table 3:** Targets gain/loss by SNPs in mRNA 3’UTRs

**Accession**	**Gene name**	**SNP position in 3’UTR**	**SNP**	**MiRNA targeting wild-type gene**	**MiRNA targeting SNP-type gene**	**Target gain/lost**
AB042609	C1rs-A	221	U → C	s0016-5p	-	Loss
AJ292212	cyp c 1.02	184	U → C	-	s0008-5p	Gain

## Discussion

The aim of this study was to predict miRNA targets in common carp and to *in silico* examine the effects of SNPs on miRNA biogenesis and target binding. Recently Yan et al. [52] identified 188 common carp miRNAs by sequencing sRNA library built from the muscle tissue. Our dataset only covered 34 of the miRNAs in their dataset. The difference in the two datasets is mainly because Yan et al. aligned the sequencing reads to the zebrafish genome to predict the miRNAs, while we used common carp sequences to identify the common carp miRNAs. The researchers did not systematically characterize the common carp miRNAs nor did they attempt to predict the miRNA targets. In the present study, we identified miRNAs and predicted the miRNA targets which will make it into a useful resource for studying miRNA function.

In our study, we classified the conserved miRNAs shared by other animals into different groups to help us investigate the evolution of these miRNAs during the divergence of the animals. We found that many of the miRNAs were conserved among several animals, suggesting that they may have essential biological functions. The conservation of these miRNAs may help us infer the functions of these miRNAs in common carp based on their known functions in other species. We discovered six conserved miRNA families in fish that are being reported here for the first time. Two of the miRNA families detected in the common carp were conserved only in fish, indicating that these miRNAs might be involved in the adaption to the aquatic environment. Strikingly, 21 miRNAs are found only in common carp. It is possible that these miRNAs are involved in regulatory interactions during adaptation to the common carp specific environment.

In this study, we focused on the identification and characterization of miRNAs in common carp. Recently, many studies identified miRNA-offset RNAs (moRNAs) generated from sequences immediately adjacent to the miRNA and miRNA* [53,54], although their functional role remains to be elucidated and these sRNAs had no obvious sequence or structural features. Indeed, after aligning cleaned sRNA sequencing reads to miRNA precursors using BLASTN with an identity value of 100%, we found that the non-miRNA regions in five pre-miRNA loci had matched sRNA reads adjacent to miRNAs, indicating that these sRNAs might be moRNAs Additional file 13: Figure S6. Another class of miRNA variant is isomiRs, which have variations with respect to the reference miRNAs [55]. These isomiRs are mainly generated by either a shift of Drosha and Dicer in the cleavage site or nucleotide additions at the 3’ end [56]. In our sRNA dataset, we also found that 1,053 sRNAs were identical to the identified miRNAs with either longer or shorter sequences, suggesting that these sRNAs are putative isomiRs.

MiRNA target identification is important to predict the functions of the miRNAs. Although computational approaches have been widely used to predict miRNA targets, most of these methods suffer from high false positive rates [57]. In the present study, we combined the results of two popular methods to predict miRNA targets. Using a set of 59 negative examples from TarBase [58], we estimated that the false positive rates of TargetScan and PITA were 28.9% and 35.5%, respectively. Significantly, a combination of these two methods gave a false positive rate of 13.5%, much lower than by either of the methods alone Additional file 14: Figure S7. We assume, therefore, that the strategy we have applied here should have reduced the number of false positive miRNA targets to a low error rate.

Because of the limited number of publicly available common carp genes with 3’UTR information, the number of targets per miRNA that we could identify was lower than the numbers reported previously [59-62]. Even so, our prediction revealed that many of the genes were regulated by multiple miRNAs. By targeting a gene with multiple miRNAs the expression of the gene can be subtly and precisely regulated. As further information is obtained, we can expect further improvements in common carp genome annotation which will provide a better overview of its miRNAs and targets.

No common carp SNPs are currently publicly available, making the identification of SNPs in miRNAs difficult. A number of challenges including sequencing errors and post-transcriptional editing, would also lead to the sequence variability between sRNAs and reference miRNAs. To avoid artificial variability by sequencing errors, we have filtered out the low-quality reads before aligning sRNAs to reference sequences. Re-sequencing genomic regions of randomly selected miRNAs and 3’UTRs with SNPs using Sanger sequencing revealed that most SNPs were successfully detected, indicating that the sequence variability was mainly from SNPs in genomic DNA. The left undetected sequences might be from miRNA post-transcriptional editing. Many studies reported that miRNA post-transcriptional editing could alter processing of some miRNAs [63] or modulate the target specificity of the mature miRNA [64]. Therefore, it is necessary to further analyze miRNA post-transcriptional editing in common carp in the future.

We focused on the prediction of the potential effects of the SNPs in the miRNA genes on miRNA production and target selection. Two SNPs in stem or anti-stem regions of the precursors led to energy changes of over 2 kcal/mol (Table 2) might greatly change the miRNA product. Two SNPs were located in the mature miRNAs. Because of the limited number of SNPs and target 3’UTRs that were obtained, we did not find any potential SNPs that change miRNA targets. The targets of miRNAs can be altered by variations in the target sequences [64]. MiRNA target loss may increase the expression of the mRNA or protein, while target gain may repress protein expression or degrade the transcript. Here, using our pipeline we identified many SNPs in potential miRNA target sites. These SNPs will be important candidates for causal variants of common carp phenotypes.

## Conclusions

This study provides data for the identification and characterization of common carp miRNAs and their potential targets. These results will help further our understanding of common carp miRNA function in gene regulation. The study further identified SNPs in miRNAs and their target genes and the effects of these SNPs on miRNA biogenesis and function was discussed. The resource data described here will be a useful resource for the scientific community to study miRNAs function and to find SNP-associated phenotypes.

## Methods

### Experimental animals

The wild common carp that were used in the experiments were bred in the Heilongjiang Fishery Institute of the Chinese Academy of Fishery Science, or obtained from the Freshwater Fisheries Research Center of the Chinese Academy of Fishery Sciences.

For sRNA sequencing, a total of 17 common carp were maintained in out-door tanks with running fresh water at 23°C and fed a commercial diet twice daily. The fish were anaesthetized with eugenol and brain, skin, liver, muscle, spleen, head kidney, body kidney, intestine, gill and heart tissue were carefully separated and snap stored at −80°C until required for RNA extraction. Equivalent concentrations of the RNAs from the tissues of the 17 fish were pooled for sequencing.

Eggs from female adult common carp were fertilized following the procedure described by Yan et al. [52]. Common carp embryos were collected at the following developmental stages: fertilized oocytes (0 h), 72 hours post-fertilization (hpf) embryos, 1 day post-hatching (dph) larva, 5 dph larva and 10 dph larva.

For SNP validation, 20 adult common carp were randomly selected and blood DNA was extracted. Blood samples (0.5 to 1 ml) were collected in 1 ml syringes primed with EDTA anticoagulation agent. The DNA was isolated with a QIAamp DNA Blood Midi Kit (QIAGEN, Hilden, Germany) and quantified using a spectrophotometer.

All animal experiments reported in this study conformed to the Chinese Academy of Fishery Science, Beijing, guidelines for the care and use of laboratory animals, and to the National Institutes of Health Guide for Care and Use of Laboratory Animals.

### Reference sequences

We used common carp BESs and transcriptome data as reference sequences to identify miRNAs because the common carp genome is still unfinished. Previously, we published 75,744 high-quality common carp BESs with a minimum length of 50 bp that were generated after base calling and trimming for *E. coli* and vector sequences [29]. The BESs were assembled into genomic contigs using the Celera assembler [65] with default parameters. An additional 49,669 transcriptome contigs that we have reported [30] were also used as reference sequences.

### Homology-based prediction of common carp miRNAs

To identify potentially conserved miRNAs from the known mature miRNAs, we downloaded all the animal miRNAs from the miRBase release 17.0 [66] and removed identical mature miRNAs. The remaining non-redundant miRNAs were aligned against the reference sequences using NCBI’s BLASTN program. To select an appropriate identity threshold for BLASTN, we aligned the miRNAs in the same families using BLASTN and found that the minimum sequence identity among the miRNAs was 90% [see Additional file 15: Figure S8. Therefore, we set the identity threshold to predict common carp miRNAs at 90% [67]. We extracted the mapped regions and 60 bp bilateral flanking sequences from the reference sequences and then ran UNAfold [32] to predict the hairpin structures of the extracted sequences. A sequence was considered to be a candidate miRNA precursor if the hairpin structure satisfied the criteria previously described by Fu et al. [17]: (1) the minimum free energy (ΔG) is ≤ −15 kcal/mol; (2) the stem region includes at least 80% of the mature miRNA; (3) the number of allowed errors in one bulge is ≤ 18 bp; (4) the hairpin is > 53 bp long; (5) the loop region is < 22 bp; and (6) the number of mismatches between the miRNA and the anti-stem sequence are ≤ 6 bp.

### Identification of common carp miRNAs from small RNA sequencing

A sRNA library was constructed and sequenced on an illumina Genome Analyzer following the manufacturer’s protocol. The entire set of reads that was used for miRNA identification was submitted to NCBI’s Gene Expression Omnibus [GEO:GSE35131].

We used MIREAP [68] to clean the initial sequencing reads by removing poor quality reads, 5’ adapter, 3’ adapter, reads containing poly(A) stretches, and reads less than 18 bp long. To annotate the sRNAs as rRNA, tRNA, snRNA and repeats, the cleaned reads were searched against the Rfam database [69], common carp ribosomal RNAs collected from GenBank, and common carp repeats [70] using BLASTN with an e-value of 0.01. All unaligned reads were then mapped to the reference sequences for miRNA identification using SOAP [71]. The secondary structures of the matched reads were generated using RNAfold [72] with default parameters and analyzed using MIREAP.

### RT-PCR and RT-qPCR

To detect miRNA expression, total RNA was extracted from the brain, muscle and liver of three young common carp (average weight: 200 g) with Trizol Reagent (Invitrogen, Carlsbad, USA). The RNAs from these tissues were mixed together in equivalent concentrations. The total RNA was polyadenylated with poly (A) polymerase (GeneCopoeia, Rockville, USA) to add poly(A) tails at the 3’ ends of the miRNAs. Then the poly(A) miRNAs were reverse transcribed using M-MLVRTase and a unique Oligo-dT adaptor primer in an All-in-One^TM^ miRNA qRT-PCR Detection Kit (GeneCopoeia, Rockville, USA). The forward primers used in the PCR that was run on an ABI 9700 thermal cycler (Applied Biosystems, Foster City, USA), were specific to the miRNAs. The PCR products were separated on 3% agarose gel and stained with ethidium bromide.

RT-qPCR was used to validate the expression profiles of the selected miRNAs. The total RNAs from the five developmental stages were reverse transcribed as described above for the miRNAs. The RT-qPCR was performed using the ABI 7500 sequence detection system (Applied Biosystems, Foster City, USA). U6 small nuclear RNA was used as an endogenous control for the miRNAs. All reactions were run in triplicate for each gene. The relative amount of miRNA to U6 RNA was calculated using the 2 − ΔΔCT method. To differentiate the expression of the selected miRNAs and to categorize them according to their expression patterns, a heatmap chart was drawn by transforming the normalized data to a log 2 scale for visualization purpose. Hierarchical clustering was performed using the R program version 2.10.1 [73]. All primers for RT-qPCR were listed in the Additional file 16: Table S8.

### Prediction of common carp miRNA target genes

To identify the putative miRNA target genes, we downloaded 506 common carp mRNAs containing 3’UTR annotation from NCBI’s dbEST [74] and extracted the 3’UTRs. We combined the results of two popular programs, TargetScan [45] and PITA [46], to identify candidate target genes. Briefly, we ran TargetScan with the default parameters and used PITA with the following parameters: 1) a seed of 6–8 bases; 2) no mismatches; 3) up to one G:U wobble in 7- or 8-mers; and 4) ΔG low than −9 Kcal/mol. When a miRNA-target pair was predicted by both TargetScan and PITA, it was considered as a miRNA target.

RT-qPCR was also used to detect the expression pattern of four selected miRNA-transcript pairs. The total RNAs from five developmental stages were treated with RNase-free DNase (Ambion, Austin, USA) according to the manufacturer’s instructions. Reverse transcription was performed with oligo (dT) primers using the First Strand cDNA Synthesis Kit (Fermentas, Burlington, Canada). RT-qPCR was performed using an ABI 7500 Sequence Detection System (Applied Biosystems, Foster City, CA, USA) with SYBR Premix Ex Taq II (Takara, Shiga, Japan) and the 2 − ΔΔCT method. The expression of each target gene was normalized to that of beta-actin. The primers used were listed in Additional file 17: Table S9.

### SNPs in the miRNAs

Cleaned reads were aligned against the common carp miRNA precursors using MAQ software (parameters set as: -N 17; -E 0; -q 20; -e 2; -D 1000) [75] to call the SNPs. The SNPs in the pre-miRNAs were classified into four types: 1) SNPs in the mature miRNAs; 2) SNPs in the stem region but not the mature region; 3) SNPs in the loops; and 4) SNPs in the anti-stem regions. To study the effect of SNPs on miRNA biogenesis, we calculated the second structure energy of the different SNP-type precursors using RNAfold [72] and compared the energy changes between SNP-type pre-miRNAs and wild type pre-miRNAs. In addition, both TargetScan and PITA were used to scan the binding sites for type 1 miRNAs using the same parameters as we used to predict target sites in the wild-type miRNAs.

### SNPs in the mRNA 3’UTRs

The previously published common carp 454 transcriptome reads [SRA:SRA009366] were cleaned with SolexaQA package [76] and the low-quality reads including poly(A/T) sequences were filtered out [30]. The cleaned 454 reads were aligned to the 506 common carp mRNAs containing 3’UTR information using BWA [77]. Reads aligned to multiple mRNAs were discarded to avoid ambiguity and only the uniquely mapped reads were selected for further analysis. We identified SNPs in the selected mRNAs using SAMtools [78] (Q value = 20). We focused on SNPs in the 3’UTRs of the mRNAs and searched for miRNA-transcript pairs in the SNP-type 3’UTRs with all the common carp miRNAs. When a miRNA-target pair was predicted in the wild-type target by both TargetScan and PITA but was not predicted in the SNP-type 3’UTR by either TargetScan or PITA, we considered this as a target loss. Conversely, if a miRNA-target pair was predicted in the SNP-type 3’UTR target but not in the wild-type 3’UTR by either TargetScan or PITA, this was considered as a target gain.

### PCR-based validation of SNPs

Candidate SNPs including those which might interfere with miRNA biogenesis or target alteration were validated by PCR analysis using the DNA from 20 wild common carp. A total of 14 primer pairs were designed based on the flanking sequences of the SNPs. All the primer sequences are listed in Additional file 18: Table S10. The PCR products were sequenced by an ABI 3730xl genetic analyzer using standard protocols. The Sanger sequences that were obtained were aligned with the miRNA precursors or mRNA sequences using CLUSTALW [79] to identify any differences.

## Competing interests

The authors declare that they have no competing interests.

## Authors’ contributions

YPZ, WX, JTW and JTL conducted the bioinformatic analysis. YMW, XP and ZY prepared the samples. SLW took charge of small RNAs. YPZ and JTL drafted the manuscript. JTL and XWS supervised the study and assisted in manuscript preparation. All authors read and approved the final manuscript.

## Supplementary Material

Additional file 1 **Figure S1.** The overall flow of the homology-based prediction of common carp miRNAs. Click here for file

Additional file 2 **Figure S2.** Analysis of the hairpin structures of animal miRNA precursors. Click here for file

Additional file 3 **Table S1.** Information of common carp miRNAs only identified by our homology-based prediction. Click here for file

Additional file 4 **Table S2.** Information of common carp miRNAs identified by both our homology-based prediction and small RNA sequencing. Click here for file

Additional file 5 **Table S3.** Information of common carp specific miRNAs identified by small RNA sequencing. Click here for file

Additional file 6 **Table S4.** Specific miRNAs of which precursors were conserved without homologous miRNAs. Click here for file

Additional file 7 **Table S5.** Primers of the selected miRNAs for PCR. Click here for file

Additional file 8 **Figure S3.** PCR products of the selected miRNAs. Click here for file

Additional file 9 **Table S6.** Predicted miRNA targets in common carp by TargetScan and PITA. Click here for file

Additional file 10 **Table S7.** 18 miRNAs generated from the antisense strands of mRNAs. Click here for file

Additional file 11 **Figure S4.** Resequencing SNP sites in five miRNAs using Sanger sequencing. Click here for file

Additional file 12 **Figure S5.** Resequencing SNP sites in nine mRNA 3’UTRs using Sanger sequencing. Click here for file

Addition file 13 **Figure S6.** The non-miRNA regions in five pre-miRNA loci had matched sRNA reads adjacent to miRNAs. Click here for file

Additional file 14 **Figure S7.** False positive rate of the combination of TargetScan and PITA. Click here for file

Additional file 15 **Figure S8.** The sequence identity among miRNAs in the same families. Click here for file

Additional file 16 **Table S8.** Primers designed specifically for the selected miRNAs for RT-qPCR. Click here for file

Additional file 17 **Table S9.** Primers designed specifically for the selected target for RT-qPCR. Click here for file

Additional file 18 **Table S10.** Primers designed for PCR-based validation SNPsof miRNA precursors or mRNA sequences. Click here for file
