# Supplementary figures and images for: Identification of common carp (Cyprinus carpio) microRNAs and microRNA-related SNPs
Source: BMC Genomics. 2012 Aug 21;13:413. doi: 10.1186/1471-2164-13-413 (PMC3478155; doi:10.1186/1471-2164-13-413)

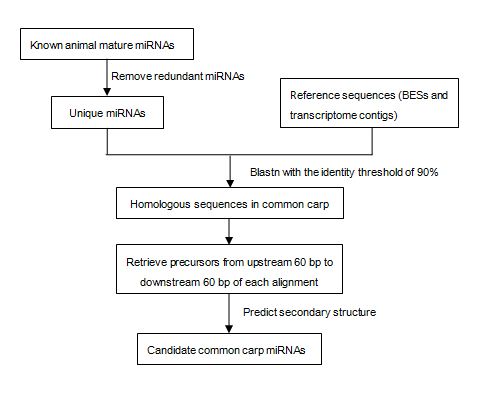

Supplement: Additional file 1 — Figure S1. The overall flow of the homology-based prediction of common carp miRNAs. [file 1471-2164-13-413-S1.png]

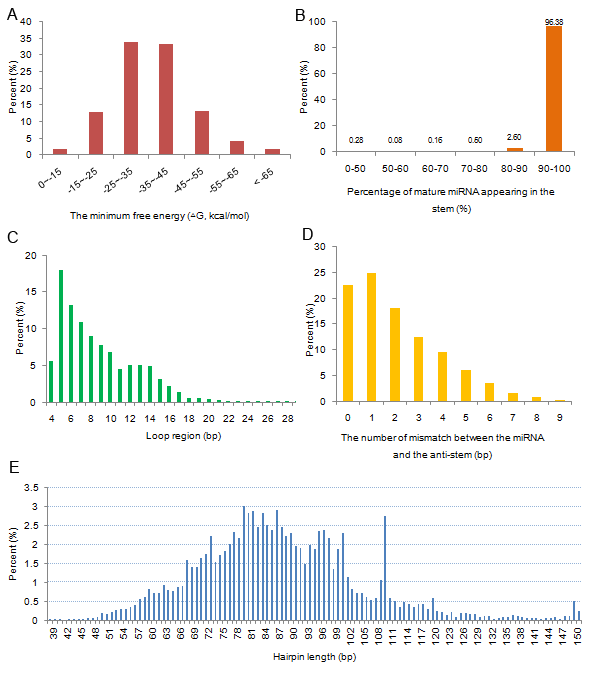

Supplement: Additional file 2 — Figure S2. Analysis of the hairpin structures of animal miRNA precursors. [file 1471-2164-13-413-S2.png]

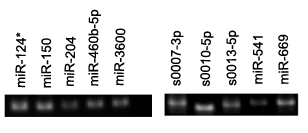

Supplement: Additional file 8 — Figure S3. PCR products of the selected miRNAs. [file 1471-2164-13-413-S8.png]

A. SNP in mir-140


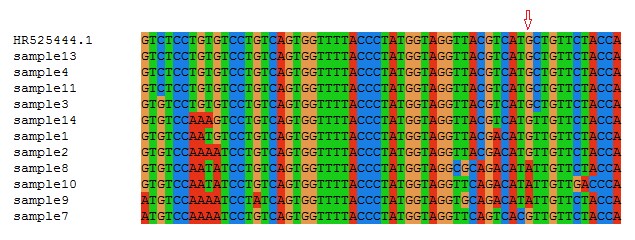


B. SNPs in s0007


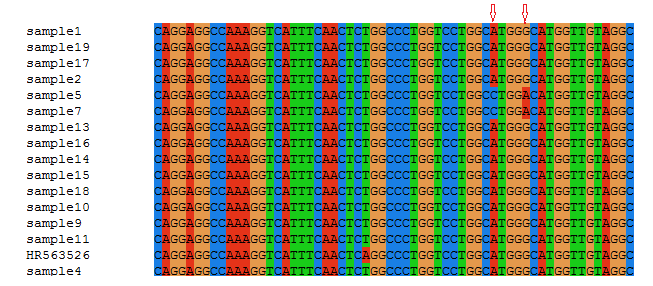


C. SNPs in s0009


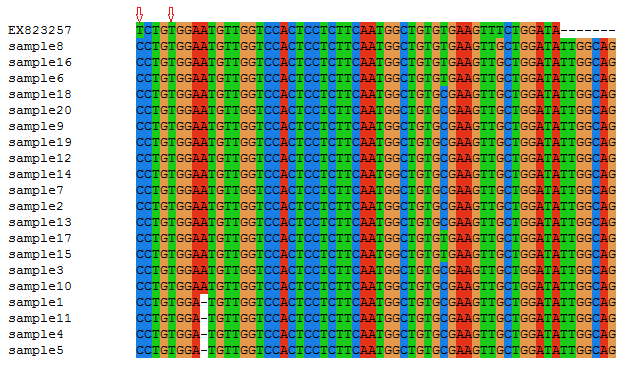


D. SNPs in s0015


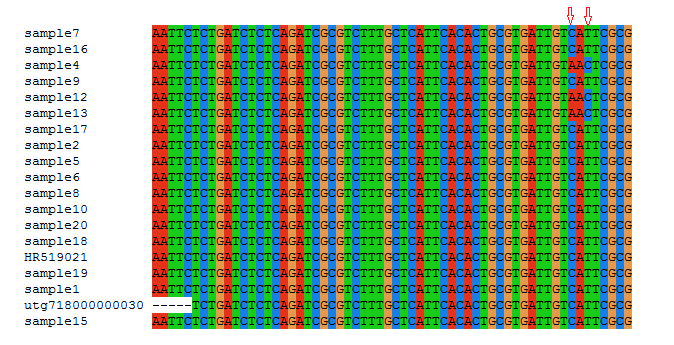


E. SNPs in s0027-1


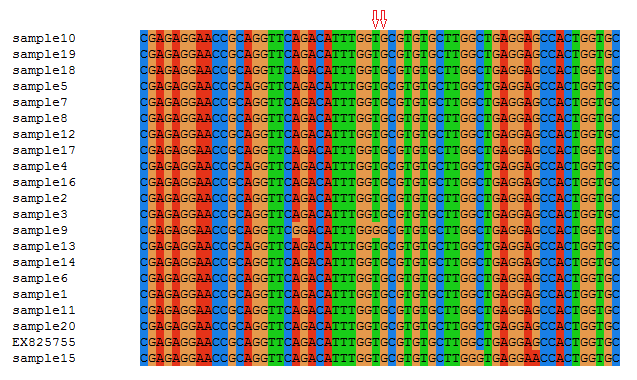

Supplement: Additional file 11 — Figure S4. Resequencing SNP sites in five miRNAs using Sanger sequencing. [file 1471-2164-13-413-S11.doc]

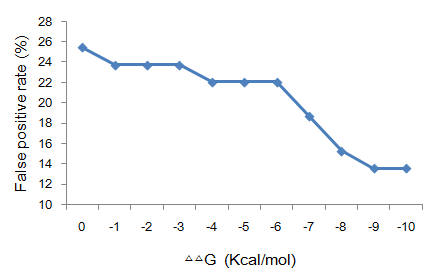

Supplement: Additional file 14 — Figure S7. False positive rate of the combination of TargetScan and PITA. [file 1471-2164-13-413-S14.png]

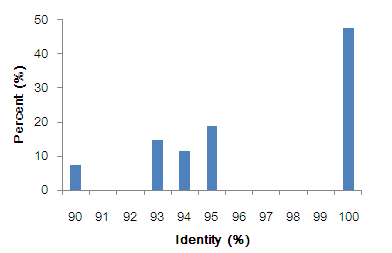

Supplement: Additional file 15 — Figure S8. The sequence identity among miRNAs in the same families. [file 1471-2164-13-413-S15.png]
