# Supplementary material for: Identification of common carp (Cyprinus carpio) microRNAs and microRNA-related SNPs
Source: BMC Genomics. 2012 Aug 21;13:413. doi: 10.1186/1471-2164-13-413 (PMC3478155; doi:10.1186/1471-2164-13-413)
Supplement: Additional file 7 — Table S5. Primers of the selected miRNAs for PCR. [file 1471-2164-13-413-S7.doc]

| **miRNA** | **miRNA specific primers** | **miRNA conservation** | **miRNA source** |
| --- | --- | --- | --- |
| miR-124* | CGTGTTCACAGCGGACCTTGAT | conserved | homology-based prediction and small RNA sequencing |
| miR-150 | TCTCCCAATCCTTGTACCAGTG | conserved | homology-based prediction and small RNA sequencing |
| miR-204 | TTCCCTTTGTCATCCTATGCC | conserved | homology-based prediction and small RNA sequencing |
| miR-460b-5p | TCCTCATTGTGCATGCTGTGTG | conserved | homology-based prediction and small RNA sequencing |
| miR-3600 | ACAGTTCTTCAGCTGGCAGCTT | conserved | homology-based prediction |
| miR-541 | TGGTGAGCGCAGAATCTGGATC | conserved | homology-based prediction |
| miR-669 | TGTGGATGTGTGCATGTACGTG | conserved | homology-based prediction |
| s0007-3p | GTGAAAGGTGTCAGGAGAAAAGCCT | conserved^※^ | small RNA sequencing |
| s0013-5p | TGGACTGAAGGTCTGTCTGCACT | conserved^※^ | small RNA sequencing |
| s0010-5p | TTGTCTGAGAGAAATTGCGCCT | specific | small RNA sequencing |

^※^ The precursors of these two miRNAs were conserved in other fish but we did not find homologous mature miRNAs in other species.
