# Supplementary material for: Identification of common carp (Cyprinus carpio) microRNAs and microRNA-related SNPs
Source: BMC Genomics. 2012 Aug 21;13:413. doi: 10.1186/1471-2164-13-413 (PMC3478155; doi:10.1186/1471-2164-13-413)

A. SNP in DQ324044 3’UTR


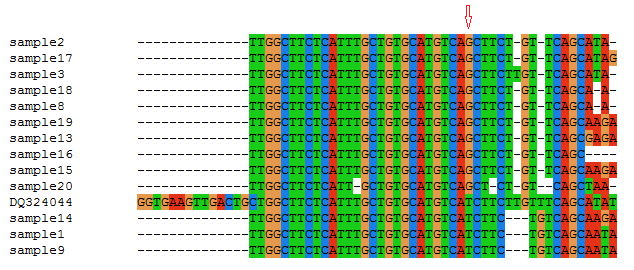


B. SNP in AB035731 3’UTR


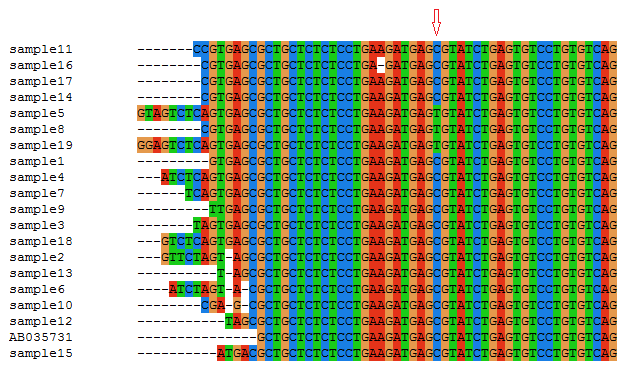


C. SNPs in AJ292212 3’UTR


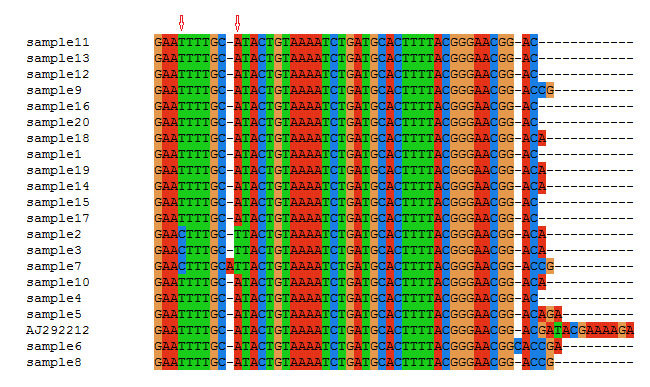


D. SNP in AB507711 3’UTR


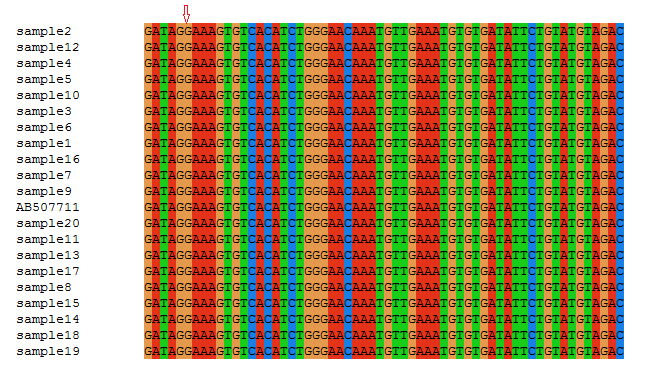


E. SNP in FJ361194 3’UTR


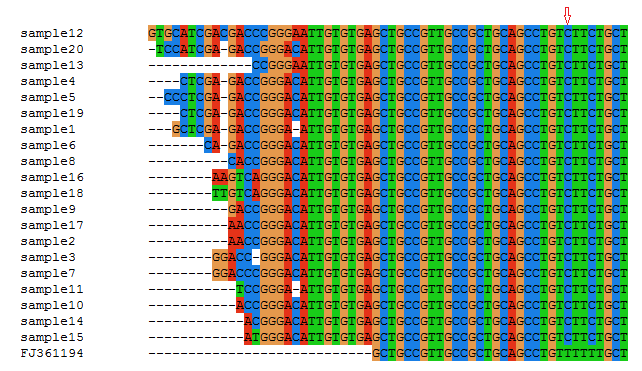


F. SNP in AY461434 3’UTR


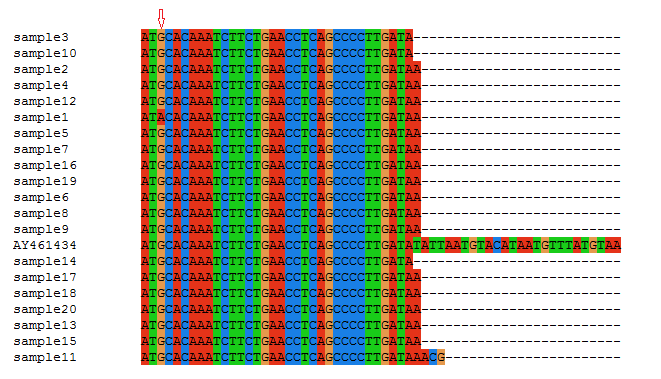


G. SNP in AY644476 3’UTR


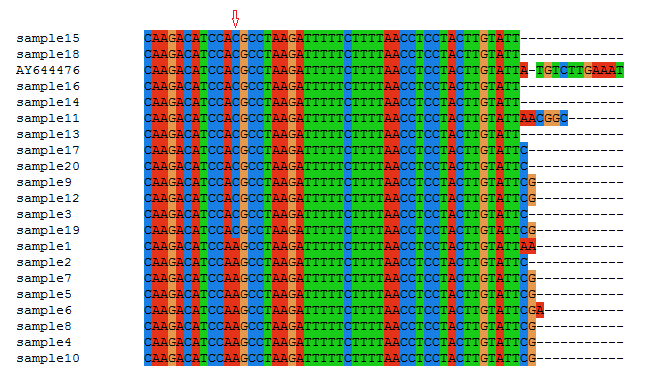


H. SNP in AB042609 3’UTR


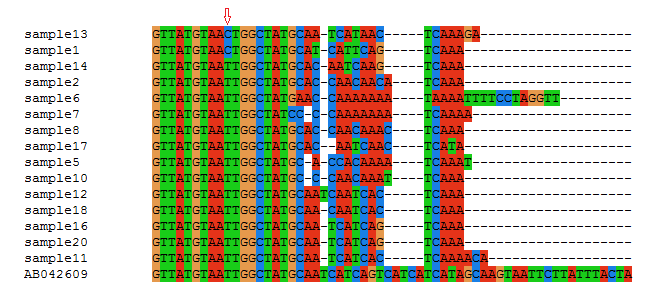


I. SNP in D85141 3’UTR


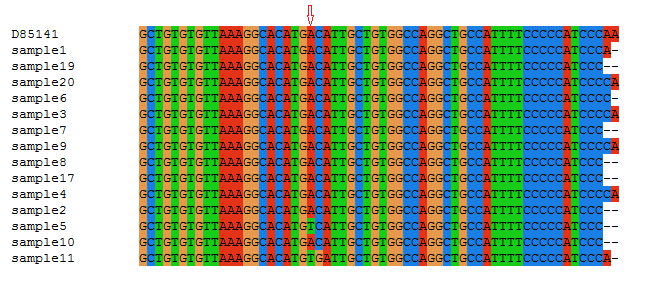

Supplement: Additional file 12 — Figure S5. Resequencing SNP sites in nine mRNA 3’UTRs using Sanger sequencing. [file 1471-2164-13-413-S12.doc]
