# Supplementary material for: Identification of common carp (Cyprinus carpio) microRNAs and microRNA-related SNPs
Source: BMC Genomics. 2012 Aug 21;13:413. doi: 10.1186/1471-2164-13-413 (PMC3478155; doi:10.1186/1471-2164-13-413)

The first sequence in each figure stands for the miRNA precursor. Two red frames show the positions of the stem region and the anti-stem region. Those sRNAs that were not fully covered in either the stem region or the anti-stem region migh be moRNAs.

A.


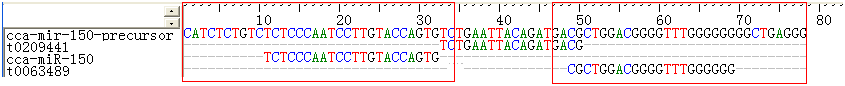


B.


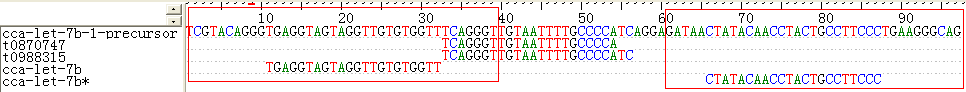


C.


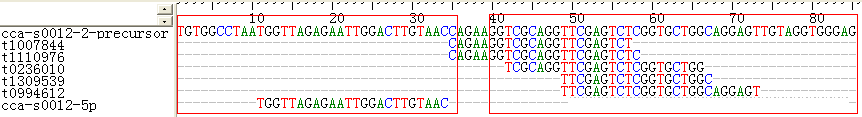


D.


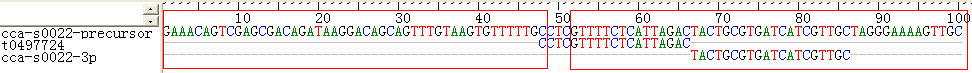


E.


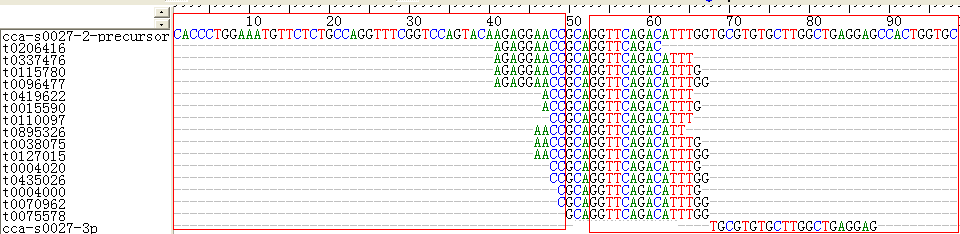

Supplement: Addition file 13 — Figure S6. The non-miRNA regions in five pre-miRNA loci had matched sRNA reads adjacent to miRNAs. [file 1471-2164-13-413-S13.doc]
