# Supplementary material for: Identification of common carp (Cyprinus carpio) microRNAs and microRNA-related SNPs
Source: BMC Genomics. 2012 Aug 21;13:413. doi: 10.1186/1471-2164-13-413 (PMC3478155; doi:10.1186/1471-2164-13-413)
Supplement: Additional file 16 — Table S8. Primers designed specifically for the selected miRNAs for RT-qPCR. [file 1471-2164-13-413-S16.doc]

| **miRNAs ID** | | **miRNA specific primers** |
| --- | --- | --- |
| miR-124* | | CGTGTTCACAGCGGACCTTGAT |
| miR-140* | | TACCACAGGGTAGAACCACGGA |
| miR-150 | | TCTCCCAATCCTTGTACCAGTG |
| miR-204 | | TTCCCTTTGTCATCCTATGCC |
| miR-22a | | AAGCTGCCAGCTGAAGAACTGT |
| miR-3065-3p | | TCAGCATCAGGATATTGTTCCC |
| miR-3065-5p | | GCAACAAAATCACTGATGCTGG |
| miR-3600 | | ACAGTTCTTCAGCTGGCAGCTT |
| miR-4453 | | AAGCTTGGTCTGGAGCGGTT |
| miR-460b-5p | | TCCTCATTGTGCATGCTGTGTG |
| miR-541 | | TGGTGAGCGCAGAATCTGGATC |
| miR-669 | | TGTGGATGTGTGCATGTACGTG |
| miR-727* | | TCAGTCTTCAATTCCTCCCAGC |
| s0007-3p | | GTGAAAGGTGTCAGGAGAAAAGCCT |
| s0007-5p | | TCATTTCAACTCTGGCCCTGGT |
| s0010-5p | | TTGTCTGAGAGAAATTGCGCCT |
| s0011-5p | | TCTAGATCTAGTGAACACGGGTGG |
| s0013-5p | | TGGACTGAAGGTCTGTCTGCACT |
| U6 | Forward | CGCTTCGGCAGCACATATAC |
|  | Reverse | TTCACGAATTTGCGTGTCA |
