# Supplementary material for: Identification of common carp (Cyprinus carpio) microRNAs and microRNA-related SNPs
Source: BMC Genomics. 2012 Aug 21;13:413. doi: 10.1186/1471-2164-13-413 (PMC3478155; doi:10.1186/1471-2164-13-413)
Supplement: Additional file 17 — Table S9. Primers designed specifically for the selected target for RT-qPCR. [file 1471-2164-13-413-S17.doc]

| **Accession** | **Forward primer** | **Reverse primer** |
| --- | --- | --- |
| AB012884 | CGGGCTTGGTAAAGTCATG | GATGGGGGGATGACGACTC |
| AB180748 | ATGATGAAGTGAACCGAATGC | TGGATGTGGTGGCGAAGT |
| EU376456 | ATGATGGAGCAGGTCGTCAAG | TCAACGCCATCAACGTTACC |
| EU499382 | GGCGAGCGGGATGTAGAGT | GTCCGATGTTCAGGATGAGG |
| beta-actin | TGCAAAGCCGGATTCGCTGG | AGTTGGTGACAATACCGTGC |
