# Supplementary material for: Identification of common carp (Cyprinus carpio) microRNAs and microRNA-related SNPs
Source: BMC Genomics. 2012 Aug 21;13:413. doi: 10.1186/1471-2164-13-413 (PMC3478155; doi:10.1186/1471-2164-13-413)
Supplement: Additional file 18 — Table S10. Primers designed for PCR-based validation SNPsof miRNA precursors or mRNA sequences. [file 1471-2164-13-413-S18.doc]

**Primers used to confirm the identified SNPs in miRNAs by using Sanger sequencing.**

| **MicroRNA precusor** | **Forward primer** | **Reverse primer** |
| --- | --- | --- |
| s0027-1 | GGATAACTGGCTTGTGGC | TAGGCGGGATTCTGACTT |
| s0009 | GCCTCACGCAGTGCAACA | TCCACGACCATCACCTCA |
| s0015 | GCGAGATGTGACAAGGAAAC | AAGTCTGCTGATTGACGAGG |
| s0007 | AGTGTCGTCATCAGGGAAGA | ATTCTCTGGTGTGTTGCTGG |
| mir-140 | TACAACAGAACCCAAGCCCT | GTAACTCTGCTCGTTGGTGT |

**Primers used to confirm the identified SNPs in targets by using Sanger sequencing.**

| **Accession** | **Forward primer** | **Reverse primer** |
| --- | --- | --- |
| DQ324044 | TGGCTTCTCATTTGCTGTGC | GTGATTCAATATGGAACGGG |
| AB035731 | AAGAGTCGCTGTTAGTCG | AGATTGAAAGATCAGCCAGT |
| AJ292212 | CAGCACACTGTCAAAACG | GTCCGTTCCCGTAAAAGT |
| AB507711 | GTCTGTCCTGTTTCTTGTATTAGTG | AAAACTCTTCTTCAGTCTACATACA |
| FJ361194 | TTTCGGCATGATTTTCAG | CTAATGCTGGGCTTTGTT |
| AY461434 | TGACAGGACGATGACGAG | TATCAAGGGGCTGAGGTT |
| AY644476 | GACCTGTAACGCTTACTCAC | AATACAAGTAGGAGGTTAAAAGA |
| AB042609 | ACATGTTCTGTGCTGGAGAT | TGACTGATGATTGCATAGCC |
| D85141 | CAGTTGACCCTCCTCGCCTTCT | GGGATGGGGGAAAATGGCAG |
